# OsASR6 Alleviates Rice Resistance to *Xanthomonas oryzae* via Transcriptional Suppression of *OsCIPK15*

**DOI:** 10.3390/ijms23126622

**Published:** 2022-06-14

**Authors:** Weiyi Guo, Songyu Chen, Youping Xu, Xinzhong Cai

**Affiliations:** 1 Key Laboratory of Biology of Crop Pathogens and Insects of Zhejiang Province, Institute of Biotechnology, College of Agriculture and Biotechnology, Zhejiang University, 866 Yu Hang Tang Road, Hangzhou 310058, China; guoweiyizju@163.com (W.G.); chensongyu1123@163.com (S.C.); 2 Centre of Analysis and Measurement, Zhejiang University, 866 Yu Hang Tang Road, Hangzhou 310058, China; ypxu@zju.edu.cn; 3 Hainan Institute, Zhejiang University, Sanya 572025, China

**Keywords:** OsASR6, *OsCIPK15*, resistance, rice, *Xanthomonas oryzae*

## Abstract

The plant-specific ASR (abscisic acid, stress and ripening) transcription factors are pivotal regulators of plant responses to abiotic stresses. However, their functions in plant disease resistance remain largely unknown. In this study, we revealed the role of OsASR6 in rice plants’ resistance to two important bacterial diseases caused by *Xanthomonas* *oryzae* pv. *oryzae* (*Xoo*) and *X.* *oryzae* pv. *oryzicola* (*Xoc*) and elucidated the mechanisms underlying OsASR6-regulated resistance. The expression of *OsASR6* was strongly elevated in response to both *Xoo* and *Xoc* challenges. Silencing of *OsASR6* in OsASR6-RNAi transgenic plants markedly enhanced rice resistance to the two bacterial pathogens. Moreover, comparative transcriptome analyses for OsASR6-RNAi and wild-type plants inoculated and uninoculated with *Xoc* demonstrated that *OsASR6* suppressed rice resistance to *Xoc* by comprehensively fine-tuning *CIPK15*- and *WRKY45-1*-mediated immunity, SA signaling and redox homeostasis. Further luciferase reporter assays confirmed that OsASR6 negatively regulated *CIPK15* but not *WRKY45-1* expression in planta. Overexpression of *OsCIPK15* strongly enhanced rice resistance to *Xoo* and *Xoc*. Collectively, these results reveal that OsASR6 alleviates rice resistance through the transcriptional suppression of *OsCIPK15*, and thus links calcium signaling to rice resistance against *X.* *oryzae*. Our findings provide insight into the mechanisms underlying OsASR6-mediated regulation of rice resistance to *X.* *oryzae*.

## 1. Introduction

Bacterial blight caused by *Xanthomonas oryzae* pv. *oryzae* (*Xoo*) and bacterial leaf streak caused by *X. oryzae* pv. *oryzicola* (*Xoc*) are devastating and economically important rice diseases [1]. Rice resistance to *Xoo* is conferred by resistance (*R*) genes. Up to now, 44 *R* genes conferring resistance to diverse strains of *Xoo* have been identified, of which 15 have been successfully cloned and characterized [2]. Unlike the resistance to *Xoo*, the rice resistance to *Xoc* seems not to comply with the gene-for-gene theory and thus is not controlled by the qualitative *R* gene. On the contrary, it is conferred by multiple quantitative trait loci (QTL) such as *qBlsr5a* and *Xo1* [3,4]. In addition, rice *WRKY45-1*, *OsMPK6*, *OsImpα1a* and *OsImpα1b* influence resistance to *Xoo* and *Xoc* [5,6,7]. The identification of novel modulators should provide more insight into the resistance of rice to these pathogens and put forward new strategies to control these rice bacterial diseases.

The abscisic acid, stress and ripening (ASR) proteins are highly hydrophilic plant-specific low-molecular-weight proteins that usually function as transcription factors (TFs) [8]. They are well-known regulators of plant responses to abiotic stresses such as drought, cold and Al tolerance [9,10,11,12]. For example, the overexpression of *OsASR1* enhances cold tolerance in transgenic rice plants [9]; *OsASR3* stands out as the best potential candidate for drought tolerance [10]; *OsASR5* mediates aluminum tolerance through STAR1 and other aluminum-responsive genes and promotes drought tolerance via a stomatal closure pathway associated with ABA and H_2_O_2_ signaling in rice [11,12]. In contrast to the well-known roles of ASRs in abiotic stress response, their functions in plants’ resistance to biotic stimuli are still not well understood. In rice, to date, only one report for *OsASR2* is publicly available. OsASR2 positively regulates the resistance to *Xoo* and *Rhizoctonia solani* through promoting expression of the defense-related gene *Os2H16* via targeting the GT-1 *cis*-element [13]. Notably, ASR exists as a small family in plants and family members may function differentially [8,10]. For example, the binding site of different OsASRs in promoters of their target genes may be distinct, GT-1 *cis*-element (GAAAAA) for OsASR2 while GGCCCAT and AGCCCAT *cis*-elements for OsASR5 [11,13]. Therefore, the functions of ASR members other than OsASR2 in rice defense against *Xoo* and *Xoc* remain to be dissected. 

This study aimed to reveal the role of OsASR6 in rice resistance to the two important bacterial pathogens *Xoo* and *Xoc* and to elucidate the mechanisms underlying this resistance. We discovered that, unlike OsASR2, which promotes rice resistance to *Xoo*, OsASR6 suppresses rice resistance to *Xoo* and *Xoc*. Furthermore, we demonstrated that OsASR6 alleviates rice resistance via transcriptional repression of *OsCIPK15*, the positive resistance modulator. 

## 2. Results

### 2.1. OsASR6 Strongly Responds to Xoc and Xoo Challenges in Rice

**Figure 1:**
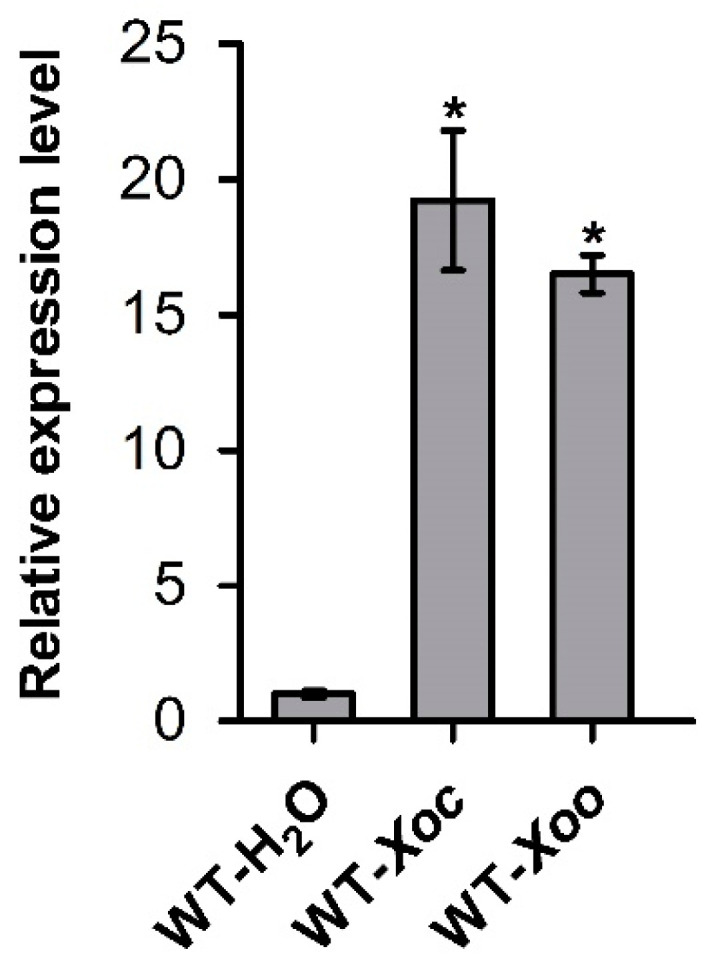
Expression of *OsASR6* is strongly induced by *Xoc* and *Xoo* inoculations. WT-H_2_O, wild-type rice plants infiltrated with sterilized H_2_O; WT-*Xoc*, wild-type rice plants inoculated by leaf infiltration with *Xoc* strain oxy04 (OD_600_ = 1); WT-*Xoo*, wild-type rice plants inoculated by leaf clipping with *Xoo* strain PXO99 (OD_600_ = 1); The data obtained at 5 dpi were shown. The quantitative real-time PCR (qRT-PCR) experiments were conducted three times, each containing three replicates. The gene relative expression level was statistically analyzed using SPSS software. Significance of the differences between mean values was determined with Student’s *t* test. Error bars indicate SD, while asterisk (*) indicates significant difference at *p* < 0.05.

To analyze the possible involvement of *OsASR6* in rice defense, we examined its expression in response to bacterial pathogens *Xoc* strain oxy04 and *Xoo* strain PXO99 in wild-type (WT) rice cultivar Nipponbare, which carries no known *R* gene conferring resistance to the above strains and thus is susceptible to them [14]. *OsASR6* expression in oxy04-inoculated rice leaves was significantly induced by 19.2 folds, compared with that in the sterilized H_2_O mock-infiltrated leaves at 5 d post inoculation (dpi) when the *Xoc*-infiltrated areas just began to show water-soaked symptoms. Likely, after clipping inoculation with PXO99, *OsASR6* expression was markedly enhanced by 16.5 folds in comparison with that in the mock-clipped control at 5 dpi when the clipping-inoculated edges initiated the necrotic symptom (Figure 1). The high sensitivity of *OsASR6* in expression to pathogen inoculations indicated that *OsASR6* is very likely involved in rice resistance to these pathogens. 

### 2.2. Silencing of OsASR6 Enhances Rice Resistance to Xoc

**Figure 2:**
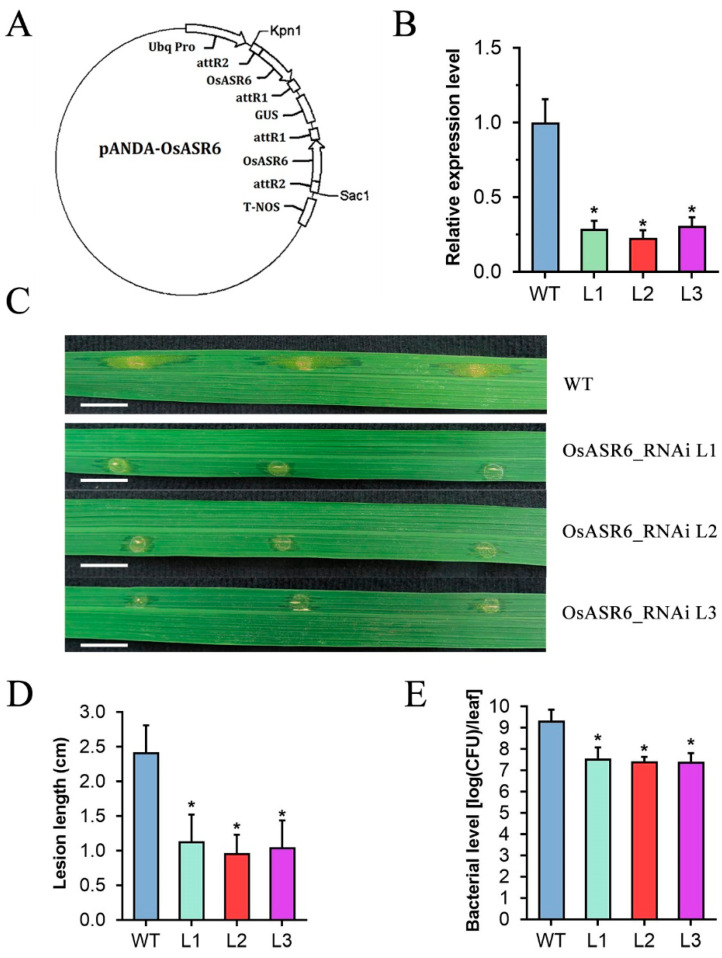
*OsASR6* strongly negatively modulates rice resistance to *Xoc*. (**A**) Schematic representation of pANDA-OsASR6 vector. (**B**) *OsASR6* gene expression of 5-week-old OsASR6-RNAi lines. (**C**) Rice bacterial leaf streak symptoms in WT and OsASR6-RNAi T_2_ lines at 10 d post inoculation (dpi) with *Xoc* strain oxy04 (OD_600_ = 1) by leaf infiltration method. Bar: 1 cm. (**D**) Statistical analysis of lesion length at 10 dpi. (**E**) Statistical analysis of bacterial level in planta at 10 dpi. Data were analyzed by Student’s *t*-test and shown as the mean ± SD. Asterisks indicate significant difference between data from OsASR6-RNAi lines (L) and those from wild-type plants (WT) (* *p* < 0.05).

To assess the function of *OsASR6* in rice disease resistance, we constructed rice transgenic lines in which *OsASR6* was silenced (OsASR6-RNAi) using *pANDA* vector based on *pUC12* with the hygromycin B resistance gene (Figure 2A). Both hygromycin resistance detection and PCR amplification for the hygromycin B gene confirmed that this gene had been integrated into the genome of the transgenic rice plants (Figure S1). Moreover, the *OsASR6* gene expression was analyzed in 5-week-old T_2_ plants using quantitative real-time PCR (qRT-PCR). As shown in Figure 2B, *OsASR6* expression was significantly suppressed in OsASR6-RNAi lines compared with that in WT plants. These results demonstrated that these OsASR6-RNAi lines are real transgenic rice plants with a suppressed expression level of the *OsASR6* gene.

To further evaluate the role of *OsASR6* in rice resistance to the important bacterial pathogen *Xoc*, we analyzed the effect of modulation of *OsASR6* gene expression on plant disease resistance. We comparatively examined the resistance of WT and the T_2_ generation of OsASR6-RNAi transgenic plants to *Xoc* strain oxy04. To obtain more accurate results, we merely conducted statistical analyses for inoculation results collected from leaves from the same positions on plants. The inoculation analyses showed that the leaves of the inoculated OsASR6-RNAi plants only generated characteristic water-soaked lesions with restricted extension, while those of the inoculated WT plants formed much larger lesions with significant extension along the veins and produced plenty of yellow bacterial oozes on the lesions (Figure 2C). The average lesion length of OsASR6-RNAi leaves of lines 1, 2 and 3 reached 1.13, 0.97 and 1.05 cm, respectively at 10 dpi, compared with 2.42 cm in leaves of WT plants (Figure 2D). Further in planta bacterial-colony counting analysis showed that inoculated leaves of OsASR6-RNAi plants only accumulated about 1.7, 1.2 and 1.2% of the bacterial amount of inoculated WT leaves at 10 dpi (Figure 2E). Taken together, these results demonstrated that *OsASR6* strongly negatively tunes rice resistance to *Xoc*.

### 2.3. Silencing of OsASR6 Promotes Rice Resistance to Xoo

**Figure 3:**
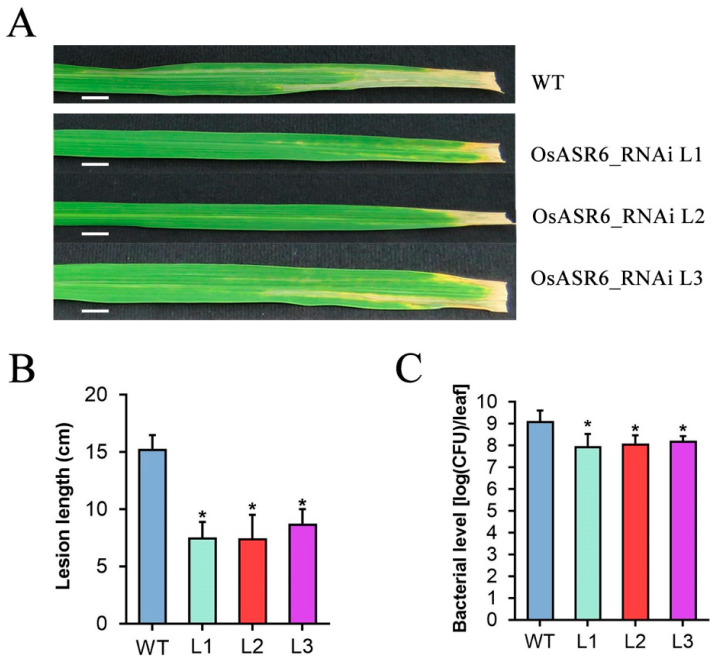
*OsASR6* negatively modulates rice resistance to *Xoo*. (**A**) Rice bacterial blight symptoms in WT and OsASR6-RNAi T_2_ lines at 14 d post inoculation (dpi) with *Xoo* strain PXO99 (OD_600_ = 1) by leaf clipping method. Bar: 1 cm. (**B**) Statistical analysis of lesion length at 14 dpi. (**C**) Statistical analysis of bacterial level in planta at 14 dpi. Data were analyzed by Student’s *t*-test and shown as the mean ± SD. Asterisks indicate significant difference between data from OsASR6-RNAi lines (L) and those from wild-type plants (WT) (* *p* < 0.05).

To further probe the role of *OsASR6* in rice resistance to another important bacterial pathogen *Xoo*, we examined the resistance of WT and the OsASR6-RNAi transgenic plants to *Xoo* strain PXO99. At 14 dpi, all three OsASR6-RNAi lines were more resistant to PXO99, with lesion lengths of 7.5, 7.5 and 8.7 cm, respectively, compared with 15.3 cm for WT plants (Figure 3A,B). Further in planta bacterial-colony counting analysis showed that inoculated leaves of OsASR6-RNAi plants only accumulated about 7.7, 8.3 and 9.8% of bacterial level of inoculated WT leaves at 14 dpi (Figure 3C). These results demonstrated that the OsASR6-RNAi lines were more resistant to PXO99 and thus *OsASR6* negatively modulates rice resistance to *Xoo*.

### 2.4. Global Transcriptome Analysis Identifies the Genes Likely Involved in Rice Resistance to Xoc

**Figure 4:**
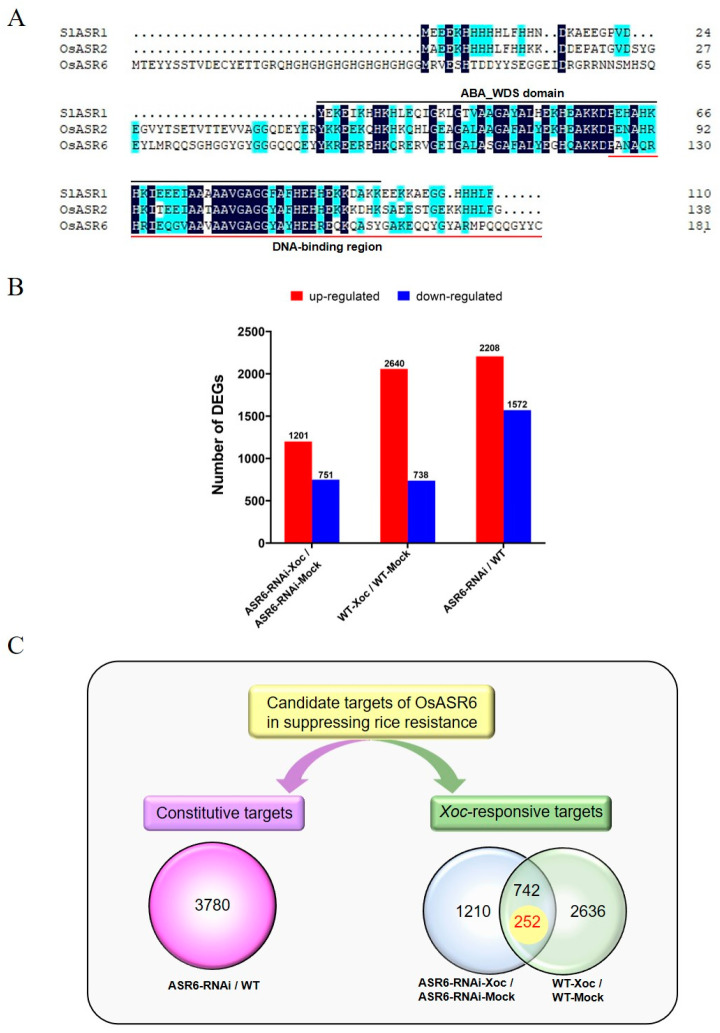
Identification of potential OsASR6 targets through transcriptome analyses. (**A**) Amino acid sequence alignment of OsASR6 with SlASR1 and OsASR2. The conserved ABA_WDS domain is overlined in black while the potential DNA-binding region is underlined in red. The identical and homologous amino acids are highlighted in blue and turquoise respectively. (**B**) Types of differentially expressed genes (DEGs). ASR6-RNAi/WT, comparison between un-inoculated OsASR6-RNAi and WT rice plants; ASR6-RNAi-Xoc/ASR6-RNAi-mock: comparison between *Xoc*-inoculated and mock-inoculated OsASR6-RNAi rice plants; WT-Xoc/WT-mock, comparison between *Xoc*-inoculated and mock-inoculated WT rice plants. (**C**) The strategies to identify constitutive and *Xoc*-responsive targets of OsASR6 function in suppressing rice resistance. Venn diagram showing the overlap between the *Xoc*-responsive genes in the WT and OsASR6-RNAi plants. The total number of overlapped DEGs is 742, among which 252 displayed reverse trend of *Xoc*-responsive expression in the WT and OsASR6-RNAi plants.

ASRs usually function as transcription factors (TFs) [8,15]. Amino acid sequence alignment of OsASR6 with SlASR1 and OsASR2 showed that OsASR6 contained the conserved ABA_WDS domain at residues 89–161 and the potential DNA-binding region at residues 124–181 (Figure 4A), which corresponds to the DNA-binding domain experimentally verified in SlASR1 [15]. Therefore, to understand the mechanisms underlying *OsASR6*-dependent modulation of rice resistance, we performed transcriptome analysis employing the OsASR6-RNAi plants. Leaves from 8-week-old WT and OsASR6-RNAi rice plants either inoculated with *Xoc* strain oxy04 or mock treated with sterilized H_2_O were harvested at 5 dpi to generate four RNA libraries for transcriptome analysis: the sterilized H_2_O mock-inoculated WT samples, the oxy04 inoculated WT samples, the sterilized H_2_O mock-inoculated OsASR6-RNAi samples and the oxy04 inoculated OsASR6-RNAi samples. The raw data from these transcriptome analyses were deposited in NCBI (PRJNA842942) and provided in the Supplemental Table S1. 

The criteria for significantly differentially expressed genes (DEGs), including significantly upregulated genes (URGs) and significantly downregulated genes (DRGs), were set as fold change >2 (for URGs) or <−2 (for DRGs) and *p* value < 0.05 in three biological replicates. Based on these criteria, a total of 3378 DEGs including 2640 URGs and 738 DRGs were identified to be responsive to *Xoc* invasion in the WT rice plants based on the comparison between WT-Xoc and WT-mock (Table S2 and Figure 4B), while 1952 DEGs including 1201 URGs and 751 DRGs were responsive to *Xoc* infection in the OsASR6-RNAi plants based on the comparison between OsASR6-RNAi-Xoc and OsASR6-RNAi-mock (Table S3 and Figure 4B). Additionally, 3780 DEGs including 2208 URGs and 1572 DRGs were affected by *OsASR6* silencing based on the comparison between OsASR6-RNAi and WT (Table S4 and Figure 4B).

Genes potentially involved in OsASR6 function in suppressing constitutive and *Xoc*-responsive rice resistance were identified from DEGs. The DEGs generated from the comparison between groups OsASR6-RNAi and WT represent the candidate targets of OsASR6 for constitutive resistance, while the DEGs exhibiting opposite abundance alteration in response to *Xoc* inoculation in WT plants (comparison WT-Xoc/WT-mock) and in OsASR6-RNAi plants (comparison OsASR6-RNAi-Xoc/OsASR6-RNAi-mock) or those differentiated merely in one of the above two comparisons represent the candidate targets of OsASR6 for *Xoc*-responsive resistance, considering that *OsASR6* strongly repressed rice resistance to *Xoc* (Figure 4C). A total of 742 genes were DEGs of both comparisons. Among them, 252 showed the opposite trend of *Xoc*-responsive expression in the WT and OsASR6-RNAi plants (Table S5 and Figure 4C). These 252 DEGs represented the import source to identify the targets of OsASR6 in *Xoc*-responsive rice resistance.

Consequently, some targets of OsASR6 in *Xoc*-responsive rice resistance were identified from the 252 DEGs. Intriguing ones included a member of the nucleotide binding site (NBS) and leucine-rich repeat (LRR) class of plant disease resistance (R) gene *PibH8* (*Pyricularia oryzae resistance b H8*), a positive resistance modulator asparagine synthetase gene *ASN1* and a negative resistance modulator protein kinase gene *CIPK5* (*Calcineurin B-like protein-interacting protein kinase 5*). *PibH8* and *ASN1* were significantly upregulated by *Xoc* inoculation in OsASR6-RNAi rice plants while strongly downregulated by *Xoc* inoculation in WT rice plants, whereas *CIPK5* exhibited the opposite expression (Table 1, Tables S2 and S3). All the above genes are involved in plant disease resistance (refer to the Discussion section) and the expression–function match results of these genes coincide well with the negative role of *OsASR6* in rice resistance to *Xoc*, demonstrating that these three genes are likely *Xoc*-responsive targets of OsASR6 in suppressing rice resistance. Other candidates included CDPK13, Annexin D4, SARD1, Cysteine-rich receptor-like protein kinase 4 and a set of genes involved in hormone signaling and redox homeostasis (Table 1).

In addition, targets of OsASR6 in *Xoc*-responsive rice resistance were also identified from the DEGs that were differentially expressed merely in one of the two comparisons (OsASR6-RNAi-Xoc/OsASR6-RNAi-mock and WT-Xoc/WT-mock) were also possible *Xoc*-responsive modulators of OsASR6-suppressive rice resistance. Some of these interesting DEGs included PAMP-induced defense regulation gene *CIPK15*, anti-*Xoc* defense negative modulator gene *WRKY45-1*, anti-*Xoo* defense negative modulator gene *WRKY76*, key gene of ethylene biosynthesis *ACS2* (*ACC synthase 2*), salicylic acid (SA) carboxymethyl transferase gene (*SAMT*), and other defense-related genes *Hsp90*, *PR1a*, *CIPK11*, *CIPK14* and *MAPKK5* (Table 1, Tables S2 and S3). Other candidates of this type are listed in Table 1.

Moreover, silencing of *OsASR6* caused 3780 genes to significantly alter their transcript abundance. Among these DEGs, which were differentially expressed in between the OsASR6-RNAi plants and the WT plants, the plant immunity activator that promotes SA production *SARD1* and jasmonic acid (JA) inducible rice PR10 *JIOsPR10* were significantly upregulated in the OsASR6-RNAi plants, while two key genes of gibberellic acid (GA) pathway *Gibberellin 20 oxidase 1* and *Gibberellin 20 oxidase 2* were remarkably downregulated in the OsASR6-RNAi plants than in the WT plants (Table 2 and Table S4). Therefore, *SARD1*, *JIOsPR10*, *Gibberellin 20 oxidase 1* and *Gibberellin 20 oxidase 2* seemed to be promising candidates of constitutive modulators of OsASR6-suppressive rice resistance, and OsASR6 might repress rice resistance via the tuning of the *SARD1*-mediated SA production, *JIOsPR10*-mediated JA pathway and *Gibberellin 20 oxidase 1*- and *Gibberellin 20 oxidase 2*-mediated GA signaling. Other candidates of this type included PibH8, ASN1, CDPKs, WRKYs, CIPKs and a set of redox homeostasis genes (Table 2).

Interestingly, some genes such as *PibH8*, *ASN1*, *WRKYs* and *CIPKs* were potential targets of OsASR6 function in suppressing both constitutive and *Xoc*-responsive rice resistance (Table 1 and Table 2). 

### 2.5. Quantitative Real-Time PCR Validation of Genes Probably Involved in OsASR6-Suppressive Rice Resistance to Xoc

**Figure 5:**
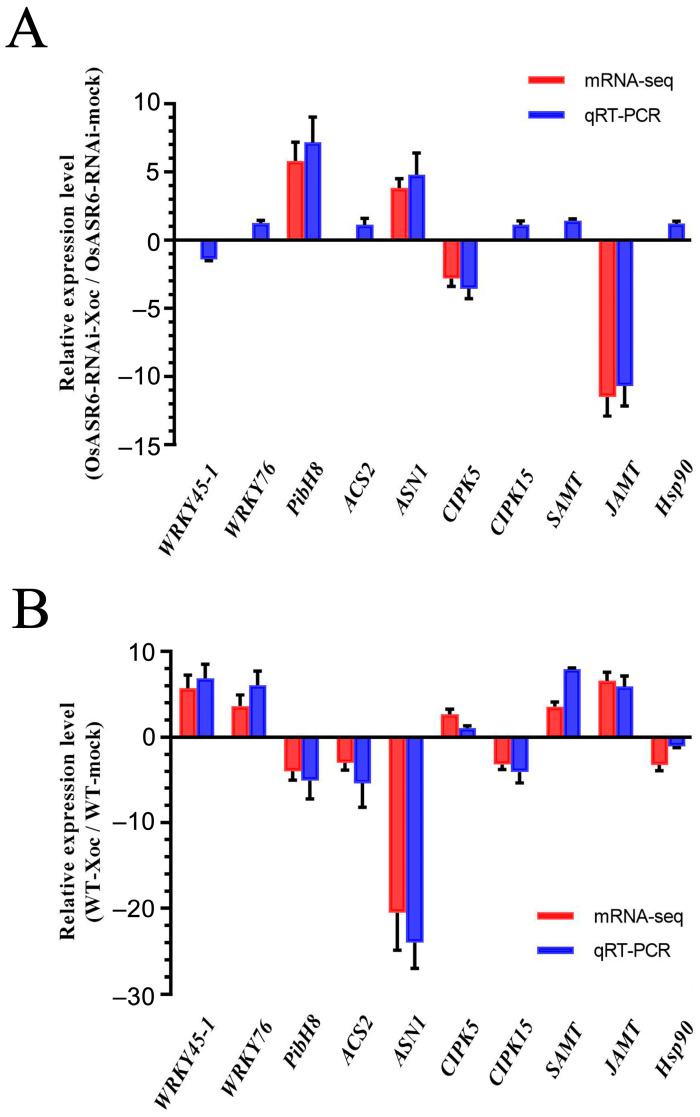
Quantitative real-time PCR validation of 10 potential OsASR6 target genes selected based on the transcriptome analysis. The relative gene expression pattern obtained from qRT-PCR experiments is shown in parallel with their gene expression pattern based on the transcriptome analysis for both comparisons WT-Xoc/WT-mock (**A**) and OsASR6-RNAi-Xoc/OsASR6-RNAi-mock (**B**). Total RNA was extracted from the Nipponbare plant leaves collected at 5 d post inoculation with *Xoc* strain oxy04 (OD_600_ = 1). The relative expression was plotted using the expression level of the *TFIIAγ5* gene as a reference. Data were shown as the mean ± SD (*n* = 3). The relative expression level for none or insignificant differential expression in the transcriptome analysis was shown as zero.

To confirm the transcriptome analysis results, quantitative real-time PCR (qRT-PCR) experiments were performed to validate the expression patterns of 10 DEGs likely involved in *OsASR6*-suppressive rice resistance to *Xoc*, including *CIPK15*, *WRKY45-1*, *ACS2*, *WRKY76*, *JAMT1*, *PibH8*, *ASN1*, *CIPK5*, *Hsp90* and *SAMT*. The results showed that the expression patterns of these 10 genes obtained from qRT-PCR analysis were generally consistent with those from the transcriptome analysis in both OsASR6-RNAi-Xoc/OsASR6-RNAi-mock and WT-Xoc/WT-mock comparisons (Figure 5). These analyses demonstrated that the results of the transcriptome analysis are reliable, and that these genes are potential targets of OsASR6 function in suppressing rice resistance.

### 2.6. OsASR6 Suppresses CIPK15 Expression in Planta

**Figure 6:**
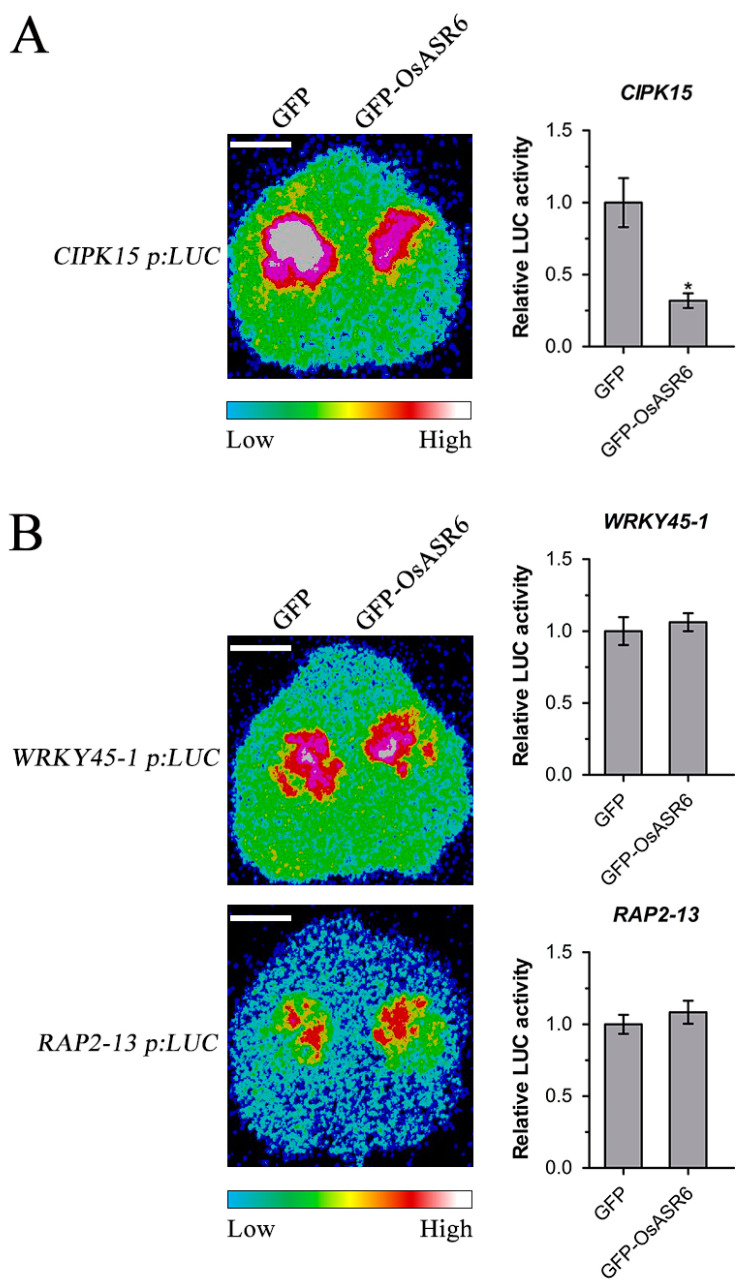
OsASR6 suppresses expression of *CIPK15* but not *WRKY45-1* and *RAP2-13* in planta. Bioluminescence of Agrobacterium-mediated co-expression of *GFP-OsASR6* (or *GFP* as control) and *CIPK15 p:LUC* (**A**) and *WRKY45-1 p:LUC* or *RAP2-13 p:LUC* (**B**) in *N. benthamiana* was detected 36 h–48 h after infiltration. Representative of three trials with similar results is shown. The bioluminescence intensity reporting the activity of luciferase (LUC) was quantified and the relative bioluminescence intensity of *GFP-OsASR6* to *GFP* is shown. Data were analyzed by Student’s *t*-test and shown as the mean ± SD. Asterisks indicate significant difference between LUC-derived bioluminescence intensity affected by GFP-OsASR6 and that by GFP (* *p* < 0.05). Bar: 1 cm.

The ASR proteins usually act as transcription factors [8]. In order to further confirm whether OsASR6 can regulate the expression of its potential target genes revealed by the transcriptome analysis in planta, we carried out transient luciferase reporter gene expression detection assays in *Nicotiana benthamiana* for three selected genes including *CIPK15*, *WRKY45-1* and *RAP2-13*. *CIPK15* was chosen since CIPKs represent important components of calcium early signaling whose function in rice resistance to *X. oryzae* remains unclear and it is the only DEG of *CIPK* type whose role in rice immunity was reported [16]. Rice *WRKY45-1* influences resistance to *Xoo* and *Xoc* [5] while *RAP2-13* is involved in ethylene signaling, which modulates rice resistance [17]. The 2.0 kb promoter of *CIPK15* normally drove the expression of the luciferase gene (*CIPK15 p:LUC*) when the GFP tag was co-expressed (Figure 6A left half of leaf). However, co-expression of *GFP-OsASR6* strongly suppressed the luciferase gene expression driven by the *CIPK15* promoter (Figure 6A right half of leaf). Quantitative analysis showed that the signal of *CIPK15 p:LUC* co-expressed with *GFP-OsASR6* was only 32% of that of co-expression of *CIPK15 p:LUC* and *GFP* (Figure 6A). These results indicated that OsASR6 suppresses *CIPK15* gene expression in planta.

The same assay was also conducted for *WRKY45-1* and *RAP2-13*, the other two putative target genes of OsASR6 revealed by the transcriptome analysis. Results showed that the 2.0 kb promoter of *WRKY45-1* and *RAP2-13* normally drove expression of luciferase gene (*WRKY45-1 p:LUC*) and (*RAP2-13 p:LUC*), respectively, when the GFP tag was co-expressed (Figure 6B left half of leaf). Co-expression of *GFP-OsASR6* did not alter significantly the luciferase gene expression driven by *WRKY45-1* and *RAP2-13* promoter, respectively (Figure 6B right half of leaf). Quantitative analysis showed that the signal of *WRKY45-1 p:LUC* or *RAP2-13 p:LUC* co-expressed with *GFP-OsASR6* was 106% and 108% of that of their co-expression with *GFP* (Figure 6B). These analyses demonstrated that OsASR6 does not directly regulate *WRKY45-1* and *RAP2-13* gene expression in planta.

### 2.7. Overexpression of OsCIPK15 Enhances Rice Resistance to Xoc

**Figure 7:**
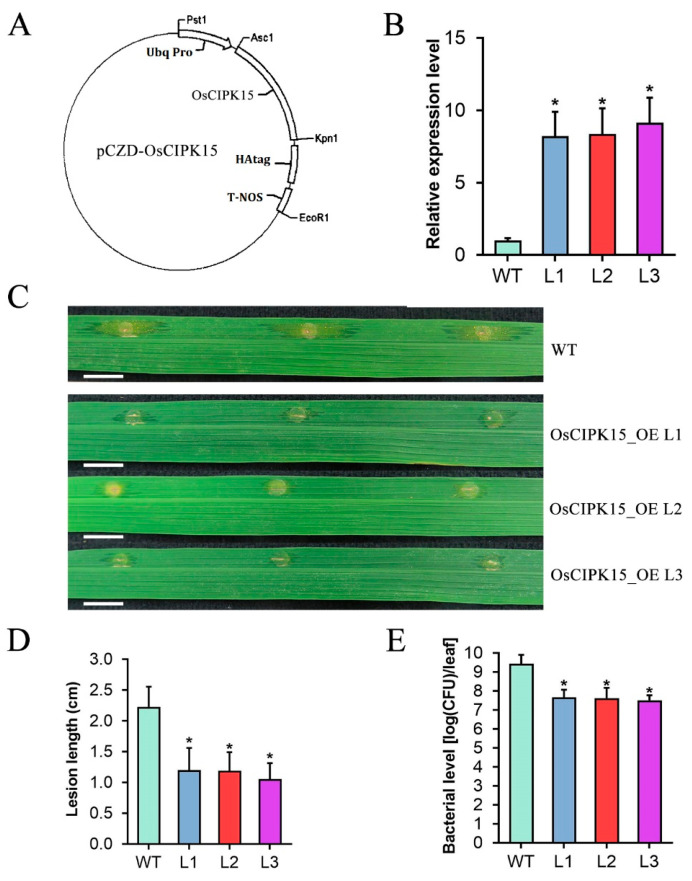
*OsCIPK15* strongly positively modulates rice resistance to *Xoc*. (**A**) Schematic representation of pCZD-OsCIPK15 vector. (**B**) *OsCIPK15* gene expression in 5-week-old OsCIPK15-OE lines. (**C**) Rice bacterial leaf streak symptoms in WT and OsCIPK15-OE T_2_ lines at 10 d post inoculation (dpi) with *Xoc* strain oxy04 (OD_600_ = 1) by leaf infiltration method. Bar: 1 cm. (**D**) Statistical analysis of lesion length at 10 dpi. (**E**) Statistical analysis of bacterial level in planta at 10 dpi. Data were analyzed by Student’s *t*-test and shown as the mean ± SD. Asterisks indicate significant difference between data from OsCIPK15-OE lines (L) and those from wild-type plants (WT) (* *p* < 0.05).

The result that OsASR6 negatively regulates *OsCIPK15* expression *in planta* prompted us to probe the function of *OsCIPK15* in rice resistance. We constructed three *OsCIPK15-*overexpression (*OsCIPK15-*OE) rice transgenic lines driven by promoter of a maize ubiquitin gene using *pCZD* vector with the hygromycin B resistance gene (Figure 7A). Both hygromycin resistance detection and PCR amplification for the hygromycin B gene confirmed that this gene had been integrated into the genome of the transgenic rice plants (Figure S2). Moreover, expression of *OsCIPK15* was analyzed in 5-week-old T_2_ plants using qRT-PCR. As shown in Figure 7B, *OsCIPK15* expression was significantly increased by over 8-fold in OsCIPK15-OE lines compared with that in WT plants. These results demonstrated that these OsCIPK15-OE lines are real transgenic rice plants with enhanced expression level of the *OsCIPK15* gene.

The confirmed T_2_ generation of OsCIPK15-OE transgenic plants were inoculated with *Xoc* strain oxy04 by bacterial infiltration. The inoculation analyses showed that the inoculated leaves of OsCIPK15-OE plants only generated characteristic water-soaked lesions with restricted extension, while the inoculated leaves of WT plants formed much larger lesions with significant extension along the veins and produced plenty of yellow bacterial oozes on the lesions (Figure 7C). The average lesion length of OsCIPK15-OE leaves of lines 1, 2 and 3 reached 1.20 cm, 1.19 cm and 1.05 cm, respectively at 10 dpi, compared with 2.23 cm in leaves of WT plants (Figure 7D). Further in planta bacterial colony counting analysis showed that inoculated leaves of OsCIPK15-OE plants only accumulated 1.7%, 1.5% and 1.2% of bacterial amount in those of WT leaves at 10 dpi (Figure 7E). Collectively, these results demonstrated that the OsCIPK15-OE lines were much more resistant to oxy04 than WT plants and thus *OsCIPK15* plays a strong positive role in rice resistance to *Xoc*. 

### 2.8. Overexpression of OsCIPK15 Increases Rice Resistance to Xoo

**Figure 8:**
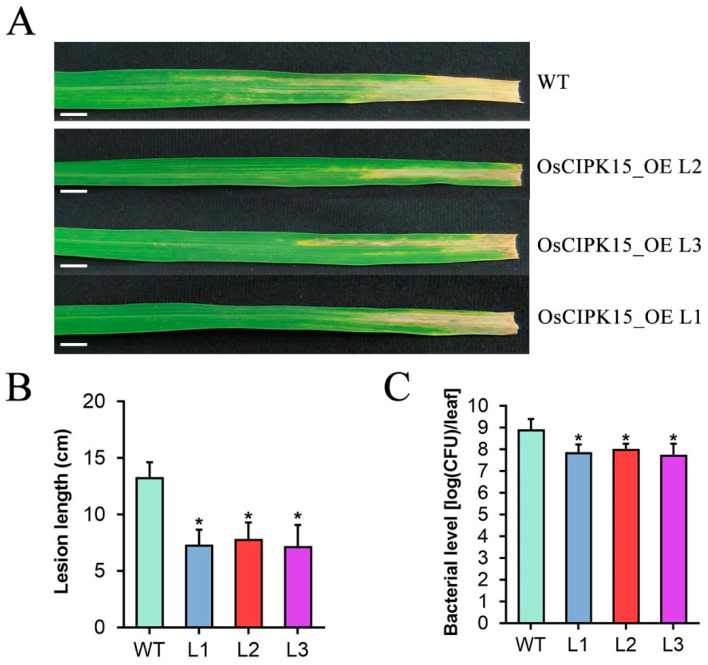
*OsCIPK15* positively influences rice resistance to *Xoo*. (**A**) Rice bacterial blight symptoms in WT and OsCIPK15-OE T_2_ lines at 14 d post inoculation (dpi) with *Xoo* strain PXO99 (OD_600_ = 1) by leaf clipping method. Bar: 1 cm. (**B**) Statistical analysis of lesion length at 14 dpi. (**C**) Statistical analysis of bacterial level in planta at 14 dpi. Data were analyzed by Student’s *t*-test and shown as the mean ± SD. Asterisks indicate significant difference between data from OsCIPK15-OE lines (L) and those from wild-type plants (WT) (* *p* < 0.05).

To further determine the role of *OsCIPK15* in rice resistance to another important bacterial pathogen *Xoo*, we examined the resistance of the OsCIPK15-OE transgenic rice plants to *Xoo* strain PXO99. At 14 dpi, all three OsCIPK15-OE lines were more resistant to PXO99, with lesion lengths of 7.3, 7.8 and 7.2 cm, respectively, compared with 13.3 cm for WT plants (Figure 8A,B). Further in planta bacterial-colony counting analysis showed that inoculated leaves of OsCIPK15-OE plants only accumulated 8.9, 12.8 and 6.9% of the bacterial amount in those of WT plants at 14 dpi (Figure 8C). These results indicated that the OsCIPK15-OE lines were more resistant to PXO99 than WT plants and thus *OsCIPK15* positively modulates rice resistance to *Xoo*.

Together with our finding that OsASR6 negatively regulates *OsCIPK15* expression in planta, these results reveal that OsASR6 alleviates rice resistance via transcriptional suppression of *OsCIPK15*, which encodes for a calcium sensor CBL-interacting protein, and thus links the calcium signaling to rice resistance against *Xoc*.

## 3. Discussion

*ASR* genes are plant-specific but broadly exist in the plant kingdom, ranging from ancient gymnosperms to angiosperms including both dicots and monocots [8]. Although their essential roles in ABA response and abiotic stress tolerance have been well known [8,9,10,11,12], the functions of ASRs in plant disease resistance remain poorly understood, which is manifested by the very limited availability of publications [8,13,18]. In this study, we provided genetic evidence to demonstrate that OsASR6 functions as a suppressor of rice resistance to both *Xoc* and *Xoo* and thus exhibits a high potential in rice molecular breeding for improved disease resistance. Previous documents have recorded the function of OsASR2 in rice resistance against *Rhizoctonia solani* and *Xoo* [13] and the roles of OsASR1, OsASR3 and OsASR5 in rice tolerance to cold, drought and aluminum [9,10,11,12]. Together, these results strongly indicate that the OsASR family plays broad and crucial roles in regulating rice responses to both biotic and abiotic stresses and thus represents excellent gene resources for rice molecular breeding for improved yields. 

Notably, OsASR6 alleviates rice resistance through transcriptional suppression of *OsCIPK15*, while OsASR2 promotes rice resistance through enhancing expression of *Os2H16* [13]. Thus OsASR6 and OsASR2 play opposite roles with distinct mechanisms in rice disease resistance. The remaining question is how these OsASRs coordinate in regulating rice disease resistance.

The mechanisms that underlie OsASR-regulated rice disease resistance remain largely unknown. The only report available to date is that OsASR2 positively regulates the resistance to *Xoo* and *Rhizoctonia solani* through promoting the expression of the defense-related gene *Os2H16* by targeting the GT-1 *cis*-element [13]. In this study, we provided evidence from transcriptome and LUC reporter expression analyses to uncover the role of OsCIPK15 in OsASR6-suppressive rice resistance to *Xoo* and *Xoc*. The contribution of CIPKs to plant resistance has previously been documented. For example, TaCIPK5 and TaCIPK10 are required for wheat resistance to stripe rust fungus *Puccinia striiformis* f.sp. *tritici* [19,20]. SlCIPK6 contributes to AvrPto-triggered immunity against *Pst* DC3000 [21]. Interestingly, OsCIPK15 plays a crucial role in the PAMPs chitin and xylanase-induced defense signaling, including hypersensitive cell death, phytoalexin biosynthesis, and pathogenesis-related gene expression in cultured rice cells [16]. Our finding that OsCIPK15 is targeted by OsASR6 in modulating rice resistance to *Xoo* and *Xoc* extends the range of CIPK in plant disease resistance. Intriguingly, CIPKs interact with the calcium sensors CBLs. Therefore, our results link the calcium signaling to rice resistance to *Xoo* and *Xoc*. In this context, it is interesting that a set of calcium-signaling components such as annexin D4, CDPK3, CDPK10, CIPK11 and CIPK14 were identified as potential candidates of the OsASR6 targets. The role of these genes in OsASR6-suppressive rice resistance awaits further study. In addition, the CBLs interacting with OsCIPK15 and the targets of OsCIPK15 in OsASR6-suppressive rice resistance need further identification. 

Our results from transcriptome and LUC reporter expression analyses demonstrate that OsASR6 directly downregulated *OsCIPK15* gene expression. Nevertheless, the binding element of OsASR6 in the promoter of *OsCIPK15* remains unidentified. The binding site of two of the six OsASRs was reported to date. OsASR2 could bind to the GT-1 *cis*-element (GAAAAA) while OsASR5 to GGCCCAT and AGCCCAT *cis*-elements [11,13]. Interestingly, three GAAAAA elements exist in the 2 kb promoter of *OsCIPK15* (Figure S3). We further conducted EMSA analyses to determine whether OsASR6 can indeed bind to the binding *cis*-elements of OsASR2 and OsASR5. The His-tagged OsASR6 was expressed in *E. coli*, affinity purified and was then used for EMSA analyses. The biotinylated 4× GGCCCAT, 4× AGCCCAT and 4× GAAAAA were used as probes, while the excessive unlabeled version of these sequences and mutated sequences were used as WT competitors and mutant competitors, respectively. One band appeared corresponding to binding of OsASR6 to labeled 4× AGCCCAT, which could be specifically completed by an unlabeled 4× AGCCCAT but not a mutant one. This type of band was also observed for 4× GAAAAA in weaker signal but not for 4× GGCCCAT probe (Figure S4). However, these bands were weak and not severely retarded (Figure S4). Therefore, whether OsASR6 binds to the AGCCCAT and GAAAAA *cis*-elements requires further verification, and comparative study on the binding specificity of the OsASR family deserves to be conducted. 

OsASR6 is a transcriptional suppressor of *OsCIPK15*. However, the expression of *OsCIPK15* decreases in the OsASR6-RNAi transgenic plants (Table 2). This should be the consequence of suppressive modulation of the *OsCIPK15* expression from repressor(s) other than OsASR6 in the OsASR6-RNAi plants, which is opposite to and stronger than the direct effect from OsASR6 on *OsCIPK15* expression. That is, the expression of *OsCIPK15* is not only modulated by OsASR6 but also affected by other factors. As a matter of fact, it was reported that hexoses, such as glucose, mannose, galactose and fructose, repressed *OsCIPK15* expression [22]. In this context, we analyzed the DEG-enriched KEGG pathways related to the hexose metabolism and found that at least 17 DEG encoding enzymes involved in the biosynthesis of hexoses including glucose, mannose and fructose through the starch and sucrose metabolism (pathway ID: ko00500) and fructose and mannose metabolism (pathway ID: ko00051) KEGG pathways were significantly upregulated in the OsASR6-RNAi plants compared with WT plants (Table S6), which is expected to lead to the accumulation of these hexoses in the OsASR6-RNAi plants. The high level of hexoses, functioning as repressors of *OsCIPK15* expression [22], causes strong reduction of the *OsCIPK15* expression in the OsASR6-RNAi plants. On the other hand, in normal conditions, the expression level of *OsASR6* is not high. Thus, in the OsASR6-RNAi plants, RNAi of *OsASR6* may only cause a moderate increase of the *OsCIPK15* expression, an effect much weaker than the repressing effect from the highly accumulated hexoses. Therefore, the net effect of the direct effect (upregulation of OsCIPK15 expression) and the indirect effect (accumulation of hexoses and thus reduction of OsCIPK15 expression) from the OsASR6 silencing in the OsASR6-RNAi plants is the decrease of the *OsCIPK15* expression. When inoculated with Xoc, however, the contribution of OsASR6 and hexoses to the *OsCIPK15* expression is reversed. The *OsASR6* expression is strongly induced by Xoc (Figure 1) and thus should be profoundly suppressed in Xoc-inoculated OsASR6-RNAi plants, which leads to the strong induction of the *OsCIPK15* expression. In contrast, most of the mentioned 17 DEGs involved in hexose biosynthesis were reduced or not significantly changed in Xoc-inoculated OsASR6-RNAi plants compared with Xoc-inoculated WT plants (Table S6). Therefore, in the Xoc-inoculated OsASR6-RNAi plants, the *OsCIPK15* expression is dominantly affected by the silencing of OsASR6 rather than hexoses and thus the Xoc-inoculated OsASR6-RNAi plants are expected to exhibit enhanced *OsCIPK15* expression compared with the Xoc-inoculated WT plants.

ASRs usually function as transcription factors (TFs) [8]. Therefore, to understand the mechanisms underlying *OsASR6*-mediated suppression of rice resistance, comprehensive transcriptome analyses for OsASR6-RNAi and wild-type plants inoculated and uninoculated with *Xoc* were performed to identify the potential transcriptional regulation targets of OsASR6. Given that OsASR6 negatively regulates rice resistance, the DEGs exhibiting opposite abundance alteration or only existing in one comparison in response to *Xoc* inoculation in WT plants (comparison between WT-Xoc and WT) and in OsASR6-RNAi plants (comparison between OsASR6-RNAi-Xoc and OsASR6-RNAi) represent the highly promising candidate targets of OsASR6. Consequently, 252 DEG genes showing opposite expression trends in response to *Xoc* inoculation in the WT and OsASR6-RNAi plants were identified (Table S5 and Figure 4C). These 252 genes represented the import source to identify the *Xoc*-responsive modulators of OsASR6-suppressive rice resistance. A large body of them are functionally unknown genes. However, interestingly, some genes have been reported to be involved in plant disease resistance. Besides *CIPK15*, these genes include *WRKY45-1*, *WRKY76*, *ACS2*, *PibH8*, *ASN1*, *CIPK5* and *SAMT* (Table 1). 

Among the list of interesting genes, *WRKY45-1* negatively modulates rice resistance to *Xoc* [5]; *WRKY76* negatively regulates rice resistance to rice bacterial blight and rice blast [23]; *ACS2* is the key gene of ethylene biosynthesis and positively modulates rice basal disease resistance [17]; *PibH8* is a member of the NBS-LRR class of plant R genes that confers rice resistance to rice blast [24]; ASN1 synthesizes asparagine from glutamine, while glutamine negatively regulates rice resistance to rice blast [25]; *CIPK5* negatively affects Arabidopsis resistance to *Pseudomonas syringae* DC3000 and *Hyaloperonospora arabidopsidis* [26]; *SAMT* negatively regulates salicylic acid (SA) thereby influencing resistance in Arabidopsis [27]. Therefore, screening out the targets of OsASR6 from the above genes remains a valuable task to be accomplished. To do so, the direct binding and gene expression regulation by OsASR6 and function in resistance to *Xoo* and *Xoc* need to be analyzed.

## 4. Materials and Methods

### 4.1. Plant Materials and Growth Conditions

The rice (*Oryza sativa*) Japonica transgenic plants used in this study were constructed in Nipponbare ecotype background by Wuhan BioRun Biosciences Co., Ltd. (Wuhan, China). Rice plants were grown in a growth room providing illumination of ~800 μmoles/m^2^/s with a 16 h light/8 h dark photoperiod at 26–28 °C. *Nicotiana benthamiana* were grown in a plant growth room providing illumination of ~300 μmoles/m^2^/s at 23–25 °C with a 16/8 h day/night photoperiod. Seeds were stored at seed storage chamber at 4 °C.

### 4.2. RNA Isolation and Quantitative Real-Time PCR Analysis

The whole rice leaves were collected from 8-week-old plants after treatments at the indicated time points and total RNA was isolated by Trizol reagent (TAKARA, Dalian, China) following the instructions provided by the manufacturer. After DNA removal using DNase I (Vazyme, Nanjing, China), the RNA samples were reverse transcribed into cDNA using HiScript Ⅱ QRT SuperMix (Vazyme, Nanjing, China). The obtained cDNAs were used for gene expression detection analysis with quantitative real-time PCR (qRT-PCR). qRT-PCR was carried out using ChamQ SYBR qPCR Master Mix (Vazyme, Nanjing, China) according to the manufacturer’s instructions on a StepOne Real-Time PCR System (Applied Biosystems, Waltham, MA, USA). The following PCR program was used: 95 °C for 30 s, 95 °C for 5 s, and 60 °C for 45 s for 40 cycles, followed by a melting-curve program. Expression of a gene-of-interest was normalized by that of rice *TFIIAγ5* gene (AK065182), which is not transcriptionally affected by the pathogen inoculation [28]. Relative gene expression values were calculated using the 2^−ΔΔCt^ method [29]. Sequences of primers used for qRT-PCR analysis are listed in Supplemental Table S7. Experiments were repeated three times. Data were statistically analyzed using SPSS software. Significance of the differences between the mean values of three independent experiments was determined with Student’s *t* test (*p* < 0.05).

### 4.3. Pathogen Inoculation and Plant Resistance Analyses

Inoculum of the bacterial pathogen *Xanthomonas oryzae* pv. *oryzae* (*Xoo*) PXO99 was prepared as described [30]. Briefly, PXO99 was grown at 28 °C in nutrient agar (NA) containing the following reagents in g/L: sucrose, 10; polypeptone, 5; yeast extract, 1; beef extract, 3; and Bacto agar, 15; pH 7.0–7.2. Single colonies were transferred to liquid NA medium with agitation until its OD_600_ approached 0.5. The bacterial cells were collected and resuspended in ddH_2_O with an OD_600_ of 1.0. Eight-week-old rice plants with fully expanded leaves were inoculated using the leaf clipping method [31]. For *X. oryzae* pv. *oryzicola* (*Xoc*) oxy04, after single colony propagation culture in NA medium, the bacterial cells were collected and resuspended in ddH_2_O. The bacterial suspension was then infiltrated into fully expanded 8-week-old rice leaves with a sterilized needleless syringe. *Xoo* and *Xoc* inoculum with an OD_600_ of 1.0 was used for inoculation and symptoms were scored by measuring lesion length. After inoculation, plants were maintained in plant growth room with a 16/8 h light/dark photoperiod at 28–30 °C. Bacterial number counting in inoculated leaves was determined as reported [32].

The inoculation analysis was performed three times, each in at least 6 plants for each treatment and gene backgrounds. Data of the lesion length and bacterial number were statistically analyzed using SPSS software. Significance of the differences between the mean values of three independent experiments was determined with Student’s *t* test (*p* < 0.05).

### 4.4. Transcriptome Sequencing

Leaf samples from 8-week-old WT and OsASR6-RNAi rice plants (ssp. Japonica cv. Nipponbare) with or without *Xoc* strain oxy04 inoculation at 5 dpi were collected and immediately frozen in liquid nitrogen. Total RNA was extracted using Trizol reagent (Invitrogen, Carlsbad, CA, USA) following the manufacturer’s procedure, and checked for quantity and purity by Bioanalyzer 2100 and RNA 6000 Nano LabChip Kit (Agilent, Santa Clara, CA, USA) with RIN value > 7.0. The qualified RNA was then converted to cDNA for transcriptome sequencing performed on an Illumina HiSeq 2500 (Illumina, San Diego, CA, USA) at the CapitalBio Technology (Beijing, China). The raw sequences were filtered to remove the adapter sequences and contaminated reads. The retained mappable reads were aligned with the HISAT software program using the default parameters [33]. The rice genome sequence ENSEMBL release-31/IRGSP-1.0.31 was used as a reference [34]. 

### 4.5. Promoter-Luciferase Gene Expression Reporter Assay in N. benthamiana

The CDS of *OsASR6* and the promotor region (2.0 kb upstream of the ATG) of *CIPK15*, *WRKY45-1* and *RAP2-13* were amplified using gene-specific primers listed in Supplemental Table S5. The CDS of *OsASR6* was inserted after the 35S promoter of the effector vector pGWB5 while the promotor region of *CIPK15*, *WRKY45-1* and *RAP2-13* was inserted before the CDS of luciferase gene in the reporter vector pGWB435, respectively, using Gateway approach. The constructs were then individually transformed into *Agrobacterium tumefaciens* strain EHA105. The *Agrobacterium* carrying one of various fusion expression vectors (effector: pGWB5-*GFP-OsASR6*; reporters: pGWB435-*CIPK15 p:LUC*, pGWB435-*WRKY45-1 p:LUC* and pGWB435-*RAP2-13 p:LUC*) was cultured overnight at 28 °C, pelleted, and resuspended to OD_600_ of 0.3 (effector) or 0.1 (reporter) in infection buffer (10 mM MES, pH 5.6, 10 mM MgCl_2_, 150 μM acetosyringone). The bacterial suspensions of effector and reporter were mixed in 1:1 ratio and incubated for at least 1 h at room temperature in dark condition. The mixture was then infiltrated into one half of newly fully expanded leaf of *N. benthamiana* plant using a needleless syringe. As a control, the other half of leaf was infiltrated with mixture of EHA105 carrying the empty effector vector with GFP tag only and a reporter construct, respectively. At 36–48 h after infiltration, the abaxial sides of leaves were spread with 1mM luciferin (Promega) and the signal was captured using a Photek camera (HRPCS5; Photek).

### 4.6. Electrophoretic Mobility Shift Assay

For generation of OsASR6 protein, the cDNA sequence of *OsASR6* was amplified through using gene-specific primers listed in Supplemental Table S5, and was then cloned into pET32a and expressed in *Escherichia coli* BL21 (DE3) pLysS (Trans-Gen Biotech). His-tagged OsASR6 protein was affinity purified and used for the electrophoretic mobility shift assay (EMSA). The biotinylated 4× GGCCCAT, 4× AGCCCAT and 4× GAAAAA probes as well as unlabeled versions of these sequences and mutated sequences were synthesized as forward and reverse strands, and were then renatured to double-stranded probes in annealing buffer [100 mM Tris-HCl, (pH 7.5), 10 mM EDTA, 1 M NaCl] at 100 °C for 5 min. The gel-shift assay was conducted according to the Thermo gel-shift assay system manual.

## Figures and Tables

**Table 1:** Potential *Xoc*-responsive targets of OsASR6 function in suppressing rice resistance.

Gene ID	Log_2_ FC Mean FPKM Value (ASR6-RNAi-Xoc/ASR6-RNAi-Mock)	Log_2_ FC Mean FPKM Value (WT-Xoc/WT-Mock)	Description
***Defense response***			
OS08G0539700	2.53 (116.69/20.15)	−2.00 (11.14/44.53)	PibH8
OS03G0291500	1.94 (7.38/1.93)	−4.36 (5.11/104.74)	Asparagine synthetase 1
OS01G0206700	−1.51 (11.32/32.23)	1.40 (6.78/2.57)	CIPK5
OS02G0627100	−1.11 (0.36/0.77)	6.53 (4.78/0.05)	Phenylalanine ammonia-lyase 1
OS03G0300400	−1.03 (1.48/3.03)	4.36 (7.10/0.35)	JIOsPR10
OS05G0127500	−1.08 (0.90/1.91)	1.25 (6.69/2.81)	SRG1
OS01G0832300	−1.76 (0.69/2.35)	1.00 (1.41/0.70)	Calcium-dependent protein kinase 3
Os10g0136500	−1.68 (3.57/11.44)	1.39 (21.76/8.28)	Cysteine-rich receptor-like protein kinase 4
OS04G0220300	−1.39 (2.26/5.90)	1.47 (3.16/1.13)	Wall-associated receptor kinase 2
OS11G0690332	−1.27 (101.37/244.27)	1.38 (265.24/101.92)	Wall-associated receptor kinase 3-like
OS01G0123900	−1.32 (78.73/196.81)	2.03 (801.36/195.99)	Bowman–Birk inhibitor 2-2
OS01G0124000	−3.28 (2.86/27.89)	3.18 (291.31/32.22)	Bowman–Birk inhibitor 2-1
OS01G0220700	−3.25 (0.59/5.61)	2.64 (1.32/0.21)	SWEET3b
OS05G0382600	−2.44 (0.50/2.70)	2.54 (11.6/2.00)	Annexin D4
OS03G0348900	1.46 (2.04/0.74)	−3.66 (1.10/13.82)	E3 ubiquitin-protein ligase MIEL1
OS07G0618000	1.86 (4.59/1.27)	−2.31 (1.12/5.85)	E3 ubiquitin-protein ligase EL5
OS07G0664600	1.78 (16.22/4.72)	−5.20 (0.50/18.31)	Momilactone A synthase
OS01G0638600	−1.85 (0.35/1.25)	1.43 (0.82/0.31)	Scopoletin glucosyltransferase
OS11G0113700	/	−1.69 (3.78/12.21)	CIPK15
OS05G0322900	/	2.51 (340.84/59.71)	WRKY45-1
OS09G0417600	/	1.85 (78.09/21.67)	WRKY76
OS04G0578000	/	−1.60 (1.78/5.39)	ACC synthase 2
OS12G0113500	/	−1.24 (6.54/15.50)	CIPK14
OS01G0824600	/	−1.46 (2.21/6.08)	CIPK11
OS12G0514500	/	−1.70 (35.94/116.94)	Heat shock protein 90
OS01G0719100	/	−1.04 (94.80/195.14)	RING zinc-finger protein 34
OS01G0699600	/	1.38 (2.73/1.05)	NPK1-related protein kinase
OS07G0129300	1.30 (87.25/35.54)	/	Pathogenesis-related gene 1a
OS03G0320600	1.07 (4.12/1.96)	/	Calmodulin-binding protein 25
OS02G0787300	1.92 (80.25/21.22)	/	Mitogen-activated protein kinase kinase 5
OS01G0160800	−1.14 (0.73/1.61)	/	Protein synthesis inhibitor I
OS11G0126100	−2.55 (0.155/0.91)	/	Protein detoxification 21
OS03G0773700	−1.88 (0.38/1.41)	/	BAM1
OS06G0587900	−1.66 (1.05/3.31)	/	EMS1
OS02G0807800	1.51 (5.57/1.96)	/	Wall-associated receptor kinase 2
OS03G0688300	1.14 (9.73/4.42)	/	Calcium-dependent protein kinase 10
***Hormone signaling***			
OS05G0102000	−3.52 (0.05/0.57)	2.73 (0.520.079)	Jasmonic acid carboxyl methyltransferase 1
OS08G0360300	−1.12 (1.33/2.90)	3.20 (3.38/0.37)	SARD1
OS08G0472800	−1.50 (0.38/1.07)	1.59 (5.53/1.84)	ABA-8′-hydroxylase 2
OS10G0371100	−2.37 (0.11/0.56)	3.14 (0.83/0.09)	RAP2-13
OS01G0883800	2.84 (15.37/2.15)	−1.70 (18.12/59.03)	Gibberellin 20 oxidase 2
OS02G0766700	/	1.24 (14.12/33.24)	b-zip transcription factor 23
OS12G0116700	/	−2.10 (0.78/3.33)	WRKY64
OS03G0758300	/	−1.17 (27.58/61.98)	CNGC2
OS01G0701700	/	1.82 (2.60/0.73)	Salicylate carboxymethyl transferase
OS02G0654700	1.42 (23.39/8.73)	/	Ethylene-responsive transcription factor 2
OS01G0190300	−1.13 (0.82/1.79)	/	Auxin-responsive protein IAA2
OS02G0643800	1.15 (8.24/3.71)	/	SAUR36
OS03G0183000	2.28 (16.66/3.44)	/	ERF073
OS03G0860100	3.32 (0.84/0.08)	/	Ethylene-responsive transcription factor 15
***Redox homeostasis***			
OS03G0348900	1.46 (2.04/0.74)	−3.66 (1.10/13.82)	Stress-related RING finger protein 1
OS01G0371200	−1.63 (0.54/1.68)	1.19 (1.40/0.61)	GSTF1
OS02G0240300	−1.01 (25.57/96.07)	1.57 (229.94/61.97)	Class III peroxidase 29
OS05G0412800	−1.44 (0.27/0.73)	1.65 (0.63/0.20)	GST 23
OS07G0677400	−1.05 (32.74/67.98)	1.17 (111.37/49.35)	Peroxidase 2
OS05G0323900	/	1.34 (302.17/119.05)	Superoxide dismutase A1
OS03G0235000	2.38 (26.95/5.19)(26.9463/5.19005)	/	Peroxidase A2

“/”: no significant difference.

**Table 2:** Potential constitutive targets of OsASR6 function in suppressing rice resistance.

Gene ID	Log_2_ FC Mean FPKM Value (ASR6-RNAi/WT)	*p* Value	Regulation	Description
***Defense response***				
OS03G0856700	2.24 (1.11/5.25)	0.00005	down	Gibberellin 20 oxidase 1
OS01G0883800	−4.68 (1.90/48.81)	0.00005	down	Gibberellin 20 oxidase 2
OS08G0360300	3.05 (2.90/0.350)	0.00005	up	SARD1
OS03G0300400	3.19 (3.03/0.33)	0.0016	up	JIOsPR10
OS01G0382000	2.13 (25.52/5.84)	0.0001	up	Pathogenesis-related gene 1b
OS01G0699600	−2.15 (0.23/1.00)	0.00945	down	NPK1-related protein kinase
OS02G0766700	−2.20 (6.89/31.65)	0.00005	down	BZIP23
OS02G0627100	3.97 (0.77/0.05)	0.0047	up	Phenylalanine ammonia-lyase 1
OS03G0291500	−5.69 (1.93/99.69)	0.00005	down	ASN1
OS01G0832300	1.81 (2.35/0.67)	0.0001	up	CDPK3
OS03G0688300	2.45 (4.42/0.81)	0.00005	up	CDPK10
OS01G0220700	4.79 (5.08/0.18)	0.00025	up	SWEET3b
OS07G0618000	−2.14 (1.27/5.57)	0.00005	down	EL5
OS01G0638600	2.10 (1.25/0.29)	0.00385	up	Scopoletin glucosyltransferase
OS03G0320600	2.17 (1.96/0.44)	0.00145	up	Calmodulin-binding protein 25
OS08G0539700	−1.07 (20.15/42.38)	0.0002	down	PibH8
OS01G0719100	−1.05 (70.48/145.72)	0.00015	down	RING zinc-finger protein 34
OS05G0322900	1.54 (165.62/57.04)	0.00005	up	WRKY45-1
OS12G0116700	1.18 (7.58/3.35)	0.00165	up	WRKY64
OS09G0417600	1.85 (74.77/20.69)	0.00005	up	WRKY76
OS11G0126100	1.61 (0.91/0.30)	0.0102	up	Detoxification 21
OS03G0773700	1.36 (1.90/0.74)	0.00605	up	BAM1
OS01G0206700	3.84 (31.82/2.22)	0.00005	up	CIPK5
OS01G0824600	−1.21 (2.46/5.68)	0.0003	down	CIPK11
OS12G0113500	−1.80 (4.22/14.67)	0.00005	down	CIPK14
OS11G0113700	−2.18 (2.56/11.63)	0.00005	down	CIPK15
OS12G0514500	−1.90 (31.12/115.85)	0.00005	down	Hsp90
OS02G0807800	2.74 (1.95/0.29)	0.00005	up	Wall-associated receptor kinase 2
OS06G0587900	2.73 (2.60/0.39)	0.00005	up	EMS1
***Hormone signaling signaling***				
OS01G0741900	−1.42 (69.90/186.71)	0.00005	down	IAA6
OS02G0723400	−1.38 (1.11/2.90)	0.0028	down	IAA8
OS02G0703600	−2.87 (2.05/15.00)	0.00005	down	ABA-8′-hydroxylase 1
OS08G0472800	−1.24 (1.10/2.61)	0.00745	down	ABA-8′-hydroxylase 2
OS04G0546800	−1.31 (65.01/161.00)	0.00005	down	Ethylene-responsive transcription factor 2
OS10G0371100	2.65 (0.56/0.09)	0.01695	up	RAP2-13
OS03G0183000	1.03 (3.44/1.69)	0.0064	up	ERF073
OS07G0664600	−1.89 (4.71/17.43)	0.00055	down	Rice microspore-preferred 8
OS05G0102000	2.93 (0.57/0.08)	0.01505	up	JAMT1
OS01G0701700	1.64 (2.19/0.70)	0.0017	up	Salicylate carboxymethyltransferase
OS04G0578000	−2.69 (0.80/5.14)	0.00005	down	ACS2
***Redox homeostasis***				
OS06G0727200	−1.71 (18.86/61.50)	0.00005	down	Catalase isozyme B
OS07G0665200	−1.50 (144.55/409.77)	0.00005	down	Superoxide dismutase 2
OS07G0694600	−2.59 (130.59/788.56)	0.00005	down	APX2
OS07G0616500	−1.34 (9.00/22.76)	0.00005	down	GLO4
OS03G0348900	−4.14 (0.74/13.14)	0.00005	down	Stress-related RING finger protein 1
OS07G0677600	−2.16 (2.01/8.96)	0.00005	down	Cationic peroxidase 1
OS01G0371200	1.47 (1.62/0.58)	0.03705	up	GSTF1
OS05G0304600	1.08 (74.34/35.16)	0.00005	up	Lipoxygenase 6
OS02G0537700	1.27 (520.38/215.89)	0.00005	up	BAS1
OS10G0536600	1.10 (1.22/0.57)	0.03295	up	Peroxidase 5

## Data Availability

Not applicable.

## Supplementary Materials

The following supporting information can be downloaded at: https://www.mdpi.com/article/10.3390/ijms23126622/s1.
