# Supplementary material for: OsASR6 Alleviates Rice Resistance to Xanthomonas oryzae via Transcriptional Suppression of OsCIPK15
Source: Int J Mol Sci. 2022 Jun 14;23(12):6622. doi: 10.3390/ijms23126622 (PMC9223573; doi:10.3390/ijms23126622)
Supplement: Supplementary file 1 [file ijms-23-06622-s001.zip › Table S7 and Figure S1-S4.pdf]

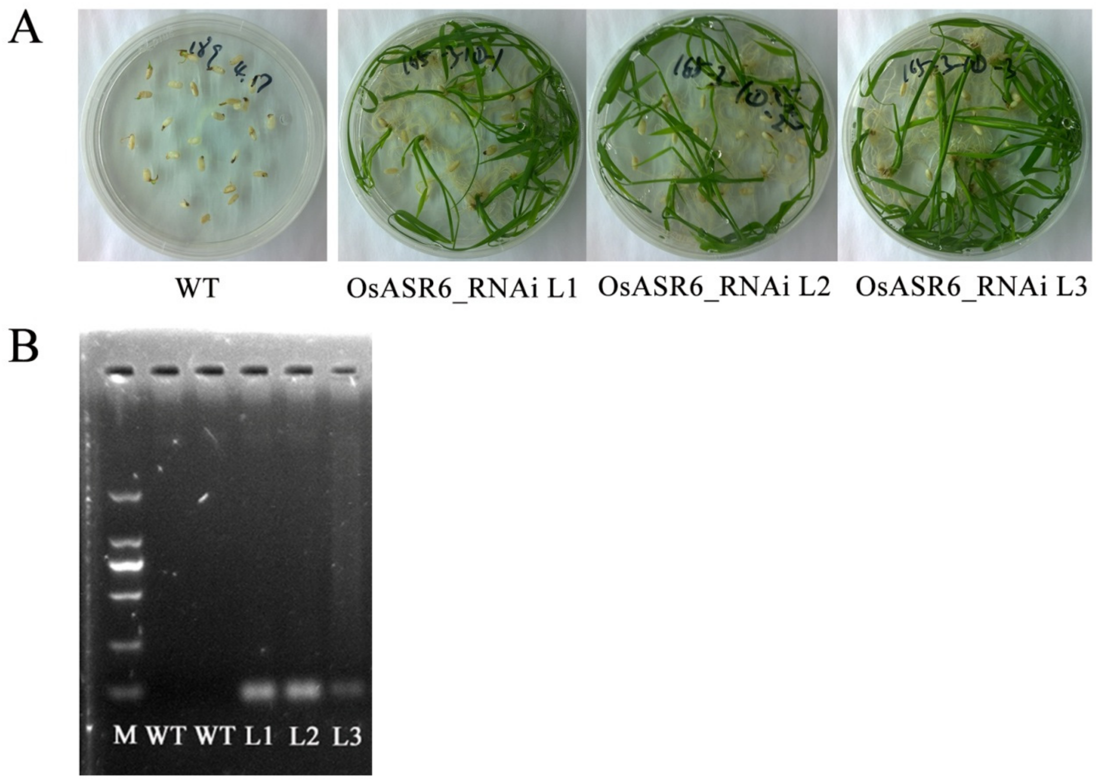

Figure S1. Verification for OsASR6_RNAi transgenic rice lines based on hygromycin detection and PCR amplification.

(A) Hygromycin resistance detection of OsASR6_RNAi lines after 14 days of sowing in 1/2 MS medium with 10 μg/ml hygromycin B.

(B) PCR amplification of hygromycin B resistance gene of OsASR6_RNAi lines. M, DL2000 DNA marker; L, OsASR6_RNAi line.

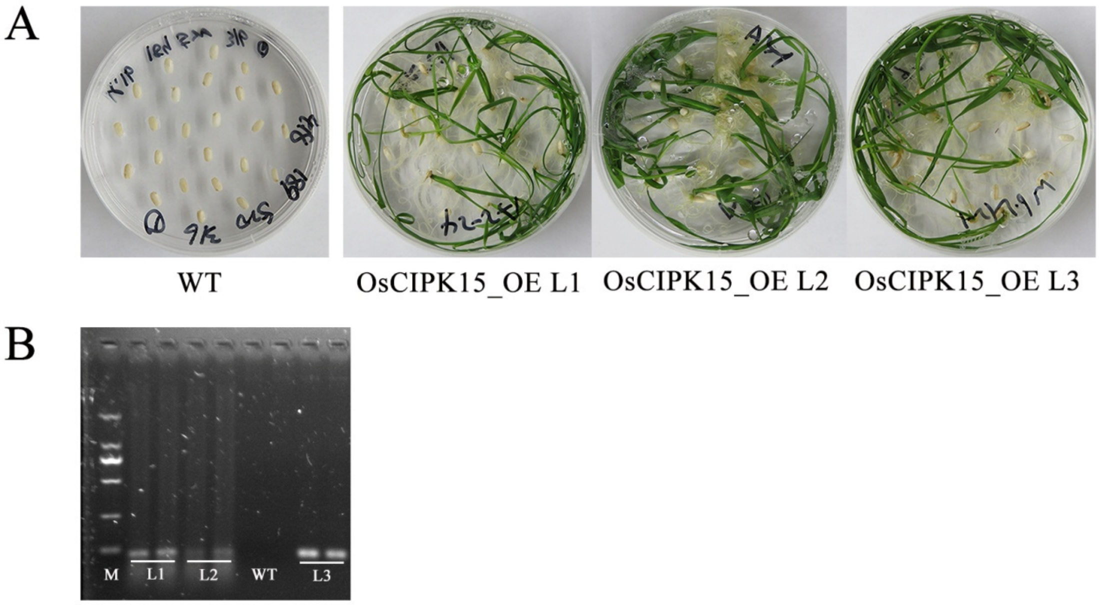

Figure S2. Verification for OsCIPK15_OE transgenic rice lines based on hygromycin detection and PCR amplification.

(A) Hygromycin resistance detection of OsCIPK15_OE lines after 14 days of sowing in 1/2 MS medium with 10 μg/ml hygromycin B.

(B) PCR amplification of hygromycin B resistance gene of OsCIPK15_OE lines. M, DL2000 DNA marker; L, OsCIPK15_OE line.

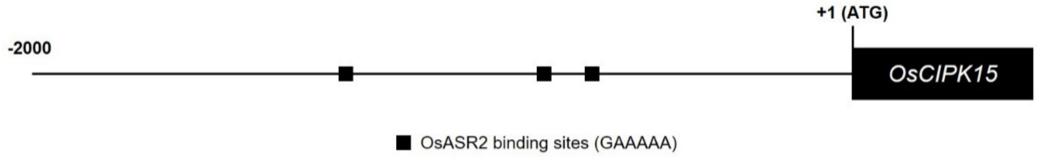

Figure S3. OsASR binding sites in *OsCIPK15* promoter.

The currently known binding *cis*-elements of OsASRs, including OsASR2 (GAAAAA) and OsASR5 (GGCCCAT and AGCCCAT), were checked in the 2 kb promoter sequence of *OsCIPK15*. Only OsASR2 binding *cis*-elements were found.

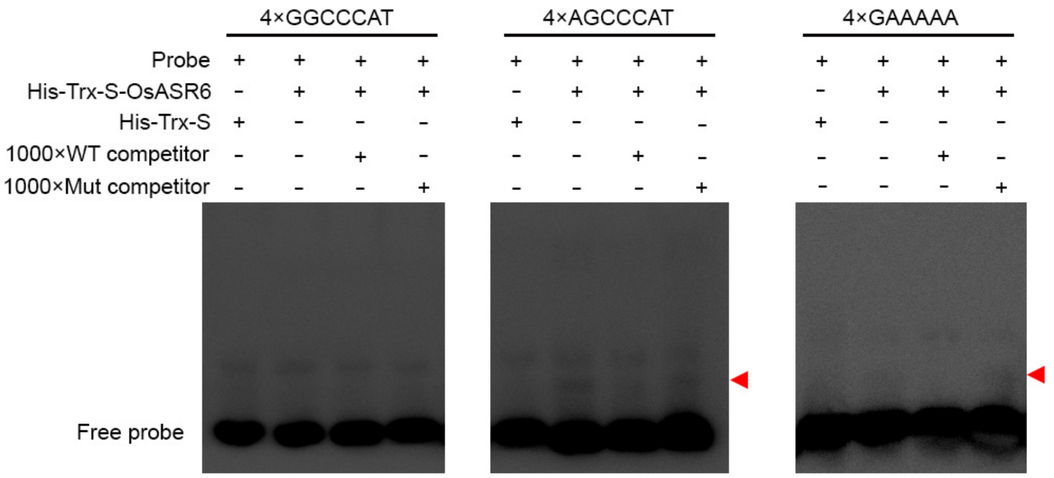

Figure S4. EMSA analyses for OsASR6.

His-tagged OsASR6 was incubated with the biotinylated 4×GGCCCAT, 4×AGCCCAT and 4×GAAAAA *cis*-elements or the excessive unlabeled version of these sequences or mutated sequences, and was then loaded and separated on a polyacrylamide gel. EMSA results were visualized by using an Odyssey infrared imaging system. Red arrows point to the bands corresponding to possible binding of OsASR6 to the probes.

Table S7. Primers and probes used in this study.

Primer /Probe Name	Sequence	Purpose
OsACS2-F	ATCATCCTTGCGTGGCTAGG	Real-time
OsACS2-R	ATTGTGCCGCAGGTCGAAC	Real-time
OsASN1-F	CATACGCATCCATCCACCGT	Real-time
OsASN1-R	GCTGCTGAGCGAGGAAGTT	Real-time
OsASR6-F	ACCACCTGATCGGAGAAGTA	Real-time
OsASR6-R	TCGCTGTAGTAGTCGTCGGT	Real-time
OsCIPK5-F	CCTTCCATTACATGGATCAGG	Real-time
OsCIPK5-R	TGGTACACCTTGGCGAAGGT	Real-time
OsCIPK15-F	GTTCTCCATCCACGATCATG	Real-time
OsCIPK15-R	CCAGATTCCTTGCATAGTGC	Real-time
OsHsp90-F	CGAATCCACGGTAGCTCTC	Real-time
OsHsp90-R	CCATTAATCACCGAGCGTGG	Real-time
OsJAMT1-F	GAGTAGTGATTAGCTAGGCG	Real-time
OsJAMT1-R	CGCTGCATGTCCGAGTTGTT	Real-time
OsPibH8-F	TGCATTGGTGGCAGAAGCAG	Real-time
OsPibH8-R	GCATCATCTCAAGCTCATCC	Real-time
OsSAMT-F	CTCTGCTAGCTAACTAGTGG	Real-time
OsSAMT-R	CTCTGAAGCGACGAGTTCTG	Real-time
OsTFIIAγ5-F	GGGTTTGCCTGGTATTTGTTAG	Real-time

OsTFIIAγ5-R	GTTGCTGCTGTGATATACTCTG	Real-time
OsWRKY45-1-F	TTGAGCTCCATCACCAGCTG	Real-time
OsWRKY45-1-R	CTTCTCCATGTCCTCCATCA	Real-time
OsWRKY76-F	TGATTACTCGAGCTCCAGAG	Real-time
OsWRKY76-R	TGGTCATCGACTTGGAATCC	Real-time
HPT-F	CTATTTCTTTGCCCTCGGACGA	PCR
HPT-R	GGACCGATGGCTGTGTAGAAG	PCR
OsASR6-LUC-F	**GGGGACAAGTTTGTACAAAAAAGCAGGCTAC**ATGACGGAGTACTACTCCAG	LUC assays
OsASR6-LUC-R	**GGGGACCACTTTGTACAAGAAAGCTGGGTC**GTTGCAGTAGTAGCCCTGCT	LUC assays
OsCIPK15-LUC-F	**GGGGACAAGTTTGTACAAAAAAGCAGGCTAC**CTTCCTCCCAGATCCGTCTA	LUC assays
OsCIPK15-LUC-R	**GGGGACCACTTTGTACAAGAAAGCTGGGTC**ACCGATCAAGAAGAGGAGAG	LUC assays
OsWRKY45-1-LUC-F	**GGGGACAAGTTTGTACAAAAAAGCAGGCTACCTAAACATGCCCAATCGGCT**	LUC assays
OsWRKY45-1-LUC-R	**GGGGACCACTTTGTACAAGAAAGCTGGGTCTGGAGGCAGGTCGGTATTTA**	LUC assays
OsRAP2-13-LUC-F	**GGGGACAAGTTTGTACAAAAAAGCAGGCTACGGTTATCGAGAATCTTGGTCC**	LUC assays
OsRAP2-13-LUC-R	**GGGGACCACTTTGTACAAGAAAGCTGGGTCATAGGCAGAGAGGGAGTAGT**	LUC assays
OsASR6-EMSA-F	**ATGACGGAGTACTACTCCAG**	EMSA assays
OsASR6-EMSA-R	**GTTGCAGTAGTAGCCCTGCT**	EMSA assays
4xGT1-F	**GAAAAAGAAAAAGAAAAAGAAAAA**	EMSA assays
4xGT1-R	**TTTTTCTTTTTCTTTTTCTTTTTC**	EMSA assays
4xGT1m-F	**TCCAAATCCAAATCCAAATCCAAA**	EMSA assays
4xGT1m-R	**TTTGGATTTGGATTTGGATTTGGA**	EMSA assays
4xASR5-1-F	**GGCCCATGGCCCATGGCCCATGGCCCAT**	EMSA assays
4xASR5-1-R	**ATGGGCCATGGGCCATGGGCCATGGGCC**	EMSA assays
4xASR5-1m-F	**GTAAAATGTAAAATGTAAAATGTAAAAT**	EMSA assays
4xASR5-1m-R	**ATTTTACATTTTACATTTTACATTTTAC**	EMSA assays
4xASR5-2-F	**AGCCCATAGCCCATAGCCCATAGCCCAT**	EMSA assays
4xASR5-2-R	**ATGGGCTATGGGCTATGGGCTATGGGCT**	EMSA assays
4xASR5-2m-F	**ATAAAATATAAAATATAAAATATAAAAT**	EMSA assays
4xASR5-2m-R	**ATTTTATATTTTATATTTTATATTTTAT**	EMSA assays
